# Heart Rate Variability Biofeedback and Mental Stress Myocardial Flow Reserve: A Randomized Clinical Trial

**DOI:** 10.1001/jamanetworkopen.2025.38416

**Published:** 2025-10-21

**Authors:** Amit J. Shah, Paolo Raggi, Hua She, Arshed A. Quyyumi, Oleksiy Levantsevych, Maggie Johnson, Kandi Schmidt, Ernest Garcia, Marina Piccinelli, Rami Abdulbaki, Naser Abdelhadi, Belal Kaseer, J. P. Ginsberg, Viola Vaccarino, J. Douglas Bremner

**Affiliations:** 1 Department of Epidemiology, Rollins School of Public Health, Emory University, Atlanta, Georgia; 2 Department of Medicine, Division of Cardiology, Emory University School of Medicine, Atlanta, Georgia; 3 Joseph Maxwell Cleland Atlanta VA Healthcare System, Decatur, Georgia; 4 Mazankowski Alberta Heart Institute, Edmonton, Alberta, Canada; 5 Department of Medicine, University of Alberta, Edmonton, Alberta, Canada; 6 Department of Internal Medicine, Trinity Health Ann Arbor, Ann Arbor, Michigan; 7 Department of Psychiatry and Behavioral Sciences, Emory University School of Medicine, Atlanta, Georgia; 8 Department of Radiology and Imaging Sciences, Emory University School of Medicine, Atlanta, Georgia; 9 Department of Pathology, George Washington University, Washington, DC; 10 Department of Medicine, West Virginia University, Morgantown; 11 Department of Psychophysiology, Saybrook University, Pasadena, California

## Abstract

**Importance:**

Heart rate variability biofeedback (HRVB) is a self-regulation intervention that targets autonomic nervous system activity through guided breathing and visualization. Investigating its effects on cardiovascular responses to psychological stress may clarify its therapeutic potential for patients with coronary artery disease (CAD).

**Objective:**

To examine the effects of HRVB on myocardial blood flow (MBF) changes during a mental stress challenge in individuals with CAD.

**Design, Setting, and Participants:**

This single-center pilot randomized clinical trial included participants aged 30 to 79 years with CAD recruited in a university hospital setting and enrolled from March 30 to November 9, 2016 (data were analyzed from January to August 2025 for final reporting). Participants were followed up for 2 months.

**Intervention:**

Six weeks of HRVB training and prescribed daily practice vs usual care (standard medical care with the intervention received after study completion).

**Main Outcomes and Measures:**

Myocardial blood flow changes after an arithmetic mental stress challenge, as measured by rubidium-82 positron emission tomography at baseline and again after 8 weeks. Linear mixed models examined the effects of HRVB on mental stress myocardial flow reserve (MFR), which is the ratio of MBF with mental stress to MBF at rest, while adjusting for baseline flow levels.

**Results:**

Data were analyzed from 21 participants (12 HRVB, 9 usual care) with mean (SD) age of 65 (6) years; 13 (61.9%) were male. In the HRVB group, the mean MFR with mental stress at baseline was 1.07 (95% CI, 0.94-1.22) and after the intervention was 1.16 (95% CI, 1.06-1.26). In the usual care group, the mean MFR with mental stress at baseline was 1.20 (95% CI, 1.05-1.38) and at the follow-up visit was 1.15 (95% CI, 0.94-1.40). While the within-group changes were not statistically significant, between-group difference in change from baseline to follow-up was significant. Specifically, the change in MFR in the HRVB group was 0.10 (95% CI, 0.01-0.19) units greater than the change in the usual care group (*P* = .03).

**Conclusions and Relevance:**

In this randomized clinical trial of participants with CAD, HRVB resulted in an increased MFR with mental stress compared with usual care. These results suggest potential benefits of HRVB during periods of mental stress and support the need for larger clinical trials to further investigate the efficacy of HRVB in cardiovascular disease prevention.

**Trial Registration:**

ClinicalTrials.gov Identifier: NCT02657382

## Key Points

**Question:**

Is heart rate variability biofeedback (HRVB) an effective intervention for reducing the effects of acute psychological stress on myocardial blood flow?

**Findings:**

In this single-center pilot randomized clinical trial of 21 participants, 6 weeks of HRVB training was associated with an increase in mental stress myocardial flow reserve, which was measured as the ratio of myocardial blood flow during mental stress to the flow at rest.

**Meaning:**

These findings suggest that 6 weeks of HRVB training is an effective intervention for increasing myocardial blood flow during an acute mental stress challenge.

## Teaser

This randomized clinical trial of patients with coronary artery disease examines whether heart rate variability biofeedback training improves myocardial blood flow reserve during a mental stress challenge.

## Introduction

Excessive psychological stress is increasingly recognized as a critical component of the pathogenesis of coronary heart disease (CHD), but despite this, stress-reduction interventions are not core components of many prevention guidelines.^1^ Studies of individuals with coronary artery disease (CAD) have reported markedly increased risk of the composite outcome of cardiovascular death and nonfatal myocardial infarction associated with mental stress–induced myocardial ischemia (MSIMI).^2^ Autonomic mechanisms related to the stress response are involved in MSIMI and have prognostic implications.^3,4^

A biobehavioral stress-reducing intervention with substantial promise is heart rate variability biofeedback (HRVB). This technique involves self-regulation of autonomic nervous system activity by training in real-time HRV augmentation through guided visualization, paced breathing, emotional awareness, and cultivation of positive emotions.^5^ HRVB has shown promise as a potential adjunct treatment for chronic stress, hypertension, and wellness improvement.^6^ Despite the previously published evidence, scientific literature lacks large-scale definitive studies involving CHD outcomes.^7^

To inform the design of a large-scale definitive study on HRVB and CHD outcomes, we conducted a pilot randomized clinical trial comparing 6 weeks of HRVB training with usual care in individuals with stable CHD. Our primary outcome was change in mental stress myocardial flow reserve (MS-MFR), which is the ratio of myocardial blood flow (MBF) during a mental stress challenge divided by the resting MBF.^8^ MS-MFR provides a continuous and sensitive measure of abnormal cardiac stress physiology. MBF reserve represents a critical physiologic parameter that reflects both microvascular and epicardial coronary function. Reduced flow reserve has been associated with increased cardiovascular events and mortality in patients with CHD, making it a clinically relevant therapeutic target.^9^ We hypothesized that MS-MFR, which results from a combination of vascular and autonomic stress mechanisms, would improve with HRVB after the 6-week intervention period compared with usual care.

## Methods

### Recruitment and Enrollment Procedures

From March 30 through November 9, 2016, this pilot randomized clinical trial recruited and enrolled participants aged 30 to 79 years with stable CAD enrolled in the observational Mental Stress Ischemia Prognosis Study who were in the follow-up phase and agreed to future research contact.^10^ All patients provided written informed consent, and the study was approved by the Emory University investigational review board. We followed the Consolidated Standards of Reporting Trials (CONSORT) guideline for randomized clinical trials.

All participants were established as patients in the extended Emory Healthcare Network that included affiliated non-Emory hospitals as previously described.^10,11^ CAD was defined based on a previous cardiac catheterization showing atherosclerosis, history of prior myocardial infarction, history of percutaneous coronary intervention or coronary artery bypass grafting at least 1 year prior to the study, or a positive nuclear stress test. Participants were excluded if they were hospitalized for acute coronary syndrome or decompensated heart failure within 1 week of the enrollment visit, if they were pregnant based on pregnancy testing, or if they had a systolic blood pressure greater than 180 mm Hg or diastolic blood pressure greater than 110 mm Hg on the day of the test. They were also excluded if they had a history of a severe mental disorder including schizophrenia, psychotic depression, bipolar disorder, or alcohol or substance dependence in the past year based on the Structured Clinical Interview for the *Diagnostic and Statistical Manual of Mental Disorders* (Fourth Edition); a history of a neurologic disorder such as dementia, stroke, or Parkinson disease; or contraindications to regadenoson administration. For each visit, β-adrenergic antagonists were withheld for 24 hours and calcium channel blockers and nitrates for at least 12 hours prior to the stress test.

After a telephone-based screening interview by the lead coordinator, participants were first randomized using a random number generator in Microsoft Excel 2016 (Microsoft Corporation) and then invited to 2 study visits at Emory University Hospital separated by 8 weeks. While the study coordinator was not blinded to treatment assignment during the time of consent, the participants were notified of their group assignment after the first visit. The HRVB group received the intervention between the first and second visits, while the usual care group received the intervention after the second visit, which we offered for incentivization.

### Sociodemographic and Health Characteristics

Participants received a clinical interview from a trained clinical coordinator who collected data on demographic history, medical history, psychiatric history, and medications. Race (Black or White), ethnicity (Hispanic), and biological sex were self-identified by the participant and collected because of their known cardiovascular significance.^12^ Other sociodemographic categories were too rare in our recruitment population to be considered for analysis. Self-reported data were also verified in participants’ electronic health records.

### Imaging Procedures

Participants underwent rubidium-82 (Rb-82) positron emission tomography (PET) cardiac imaging of the heart at rest and during a mental stress task. They were scanned on the Biograph-40 SD PET/CT (Siemens Healthineers) and received a total of 30 to 60 mCi of Rb-82 intravenously for each scan. Rest images were obtained in list mode for 7.5 minutes immediately at the beginning of the rubidium scan. Participants then underwent a 3-minute mental stress task (solving complicated mathematical problems with negative feedback).^13^ Difficulty of math problems was titrated to individual ability in order to create a stressful task. PET imaging began within 2 minutes after the mental stress task, during which time the coordinator returned to the control room and the imaging technician initiated the Rb-82 generator. The radioisotope was injected as participants underwent cardiac PET imaging. These methods have been found to have high reproducibility in terms of physiologic stress response.^13,14^

PET images were reconstructed and translated in short axis orientation scans using in-house software. Images were then transferred to the Emory Cardiac Toolbox (ECTb) 3, which contains the FlowTool for MBF calculations that were performed on summed images at 2 to 5 minutes after start of acquisition when tissue to blood contrast was high. Time activity curves were generated from regions of interest for calculation of MBF using the factor analysis method of El-Fakhri et al^15^ and the kinetic model of Hutchins et al.^16^ A model with 2 tissue compartments and 3 parameters was used to fit the data using a time-weighted least-squares figure of merit, where K1 represents the rate the tracer moves from the blood to the first tissue compartment and was interpreted as representing flow multiplied by the myocardial extraction fraction.^17^ MFR correction using equations from Yoshida et al^17^ or Lortie et al^18^ was implemented in the ECTb FlowTool. The user also had the option to apply a partial volume correction by applying a general recovery coefficient^19^ to the myocardial tissue. As per convention, MS-MFR was calculated by blinded readers (E.G., M.P.) as the ratio of total flow during mental stress to flow during rest.

The readers also calculated myocardial perfusion deficits using a 17-segment model with scores ranging from 0 (normal perfusion) to 4 (absent perfusion). In addition, we used an automated scoring system in ECTb to quantify perfusion and left ventricular ejection fraction (LVEF) in a reproducible manner.

### HRVB Intervention Description

The HRVB group underwent 6 weekly 1-hour sessions of HRVB training administered by a HeartMath LLC certified biofeedback trainer under the supervision of A.J.S and J.P.G. HRVB training was conducted in an interactive manner using a computerized system to monitor and display individuals’ HRV patterns in real time while heart rate was recorded. The first 2 visits and the last visit were in person and consisted of HRVB education and practice.

During in-laboratory training, participants were monitored with an ear-based pulse sensor and underwent guided breathing at 6 breaths per minute while also monitoring their levels of coherence between HRV and respiration variability under the supervision of the HRVB trainer. Positive emotion and interactive visual images were also introduced as methods to increase coherence. Participants were instructed to practice at home for at least 20 minutes daily and were provided with the emWave2 handheld device (HeartMath LLC) to measure their HRV during practice. The emWave2 device provides real-time feedback on participant coherence levels by measuring the low-frequency spectral HRV power using photoplethysmography signals from the finger or earlobe.^20^

The third through fifth sessions, which focused on strategies for reaching a goal of at least 20 minutes of daily practice, were performed via telephone. Participants’ progress with the handheld HRVB device was also discussed. The last session was performed in person and included live practice in which any progress or obstacles were discussed.

### Usual Care Group

The usual care group received standard medical care between the baseline and follow-up visits. To maintain participation and address ethical considerations, the usual care group was offered the HRVB intervention after completing their second study visit (poststudy completion).

### Statistical Analysis

The sample size was chosen to help design future randomized clinical trials that would follow from this pilot. We targeted an enrollment of 24 participants (12 per group), which provided sufficient statistical power (α = .05, β = 0.80) to detect effect sizes of Cohen *d* equal to 1.21 or greater.

We present the means and SDs of baseline characteristics in the HRVB and control groups. In an exploratory secondary analysis of the smaller subset of participants with available data, we compared visit and group differences in myocardial perfusion measures—specifically rest, stress, and stress-rest difference—using the mean values from 2 independent readers (P.R., E.G.) and the ECTb software. We applied similar analyses to LVEF, using *t* tests to assess differences. Data were analyzed from January to August 2025 using SAS, version 9.4 (SAS Institute Inc). We defined statistical significance as 2-sided *P* < .05.

We log-transformed MS-MFR to achieve normality, and our primary outcome was the change in log MS-MFR from visit 1 to visit 2. We used linear mixed models with random effects for individuals and fixed effects for treatment group and baseline log MS-MFR, which allowed us to account for baseline imbalances in MS-MFR by group. The model estimated the variance component associated with the individual-level random effect using restricted maximum likelihood. The significance of the random effect was assessed using a likelihood ratio test comparing the model with and without the random intercept. We used similar models for our secondary analysis of hemodynamic changes with stress.

## Results

The analytic sample included 21 participants (12 HRVB, 9 usual care) with mean (SD) age of 65 (6.0) years. Eight participants (38.1%) were female and 13 (61.9%) were male; 10 (47.6%) were Black, 11 (52.4%) were White, and none were Hispanic ethnicity.

**Figure 1.**
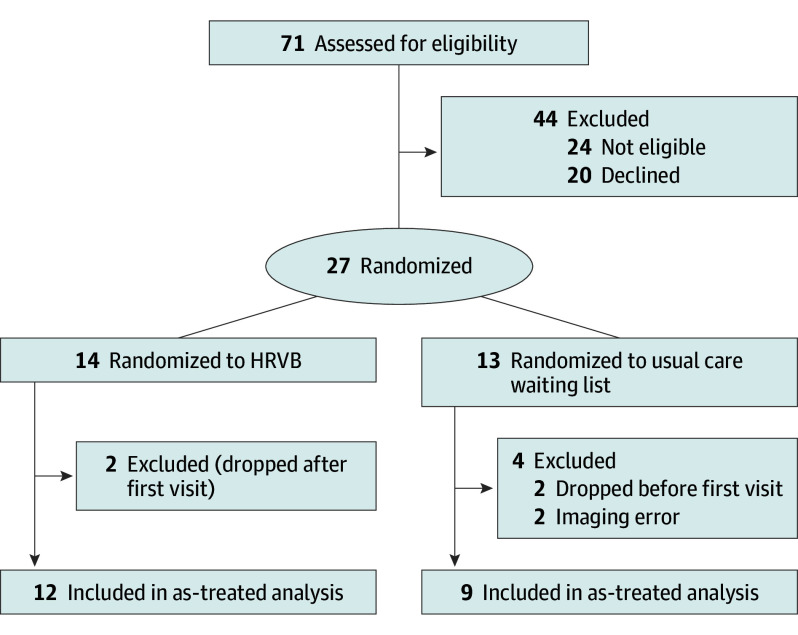
Flow Diagram HRVB indicates heart rate variability biofeedback.

We assessed 71 participants for eligibility, invited 47, and consented 27. The CONSORT diagram (Figure 1) has more details. Of the consented individuals, 14 were randomized to the HRVB group and 13 to the usual care (control) group. Two (14.3%) in the HRVB group dropped out after the first visit, and 2 (15.4%) in the control group dropped out before the first visit. Twenty-three patients finished the study; however, technician errors resulting in incorrect blood pool reference measurements limited PET data collection for 2 participants (15.4%) in the control group. We were therefore able to analyze a total of 21 patients with usable data, including 12 in the HRVB group and 9 in the control group. No serious adverse events due to the intervention or study procedures were reported.

Table 1 shows the baseline characteristics in the main analytic sample of 21 participants. Although age was similar between groups, several notable differences were observed: the HRVB group included more Black participants, a higher prevalence of tobacco smoking history, and a higher mean BMI compared with the usual care group. Conversely, a lower proportion of participants in the HRVB group had a history of coronary artery bypass grafting.

**Table 1.** Demographic Variables and Risk Factors for Patients With CAD

Characteristic	Participants (N = 21)^a^	
	HRVB (n = 12 [57.1%])	Usual care (n = 9 [42.9%])
Age, mean (SD), y	65.7 (4.7)	64.3 (7.7)
Sex		
Female	5 (41.7)	3 (33.3)
Male	7 (58.3)	6 (66.7)
Self-reported race		
Black	7 (58.3)	3 (33.3)
White	5 (41.7)	6 (66.7)
Self-reported Hispanic ethnicity	0	0
Tobacco smoking status		
Past	6 (50.0)	2 (22.2)
Current	2 (16.7)	1 (11.1)
BMI, mean (SD)	30.9 (4.4)	27.5 (4.9)
History of diabetes	3 (33.0)	4 (44.4)
BDI-II score, mean (SD)^b^	6.7 (5.1)	7.0 (5.0)
Lifetime depression	5 (41.7)	2 (22.2)
Lifetime PTSD	1 (8.3)	1 (11.1)
History of CABG	1 (8.3)	3 (33.3)
Medication use		
β-Blocker	8 (66.7)	6 (66.7)
Antidepressant	2 (16.7)	2 (16.7)

Abbreviations: BDI-II, Beck Depression Inventory II; BMI, body mass index (calculated as weight in kilograms divided by height in meters squared); CABG, coronary artery bypass surgery; CAD, coronary artery disease; HRVB, heart rate variability biofeedback; PTSD, posttraumatic stress disorder.

^a^ Data are presented as number (percentage) of participants unless otherwise indicated.

^b^ Score range, 0 to 63, with higher scores indicating more depressive symptoms.

**Figure 2.**
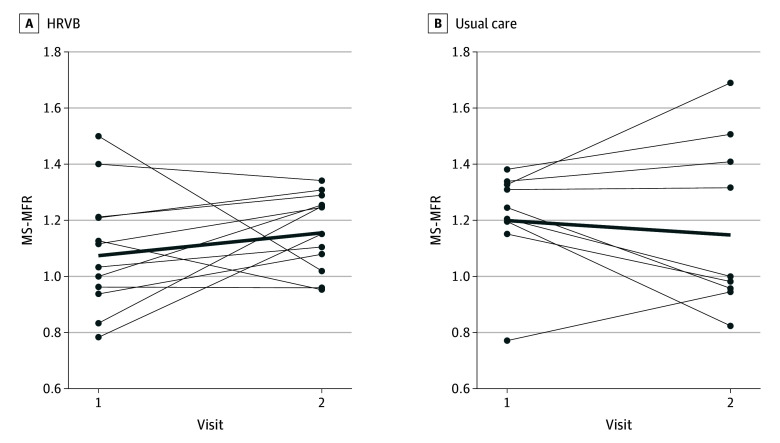
Change in the Log Mental Stress Myocardial Flow Reserve (MS-MFR) Between Visits 1 and 2 in the Heart Rate Variability Biofeedback (HRVB) and Usual Care Groups MS-MFR is measured as the ratio of myocardial blood flow during mental stress to the flow at rest. Thick bold lines represent the linear regression line for each group.

Table 2 presents MBF and MS-MFR both by cardiac territory and overall. The variance component for the individual-level random effect was highly significant, supporting the inclusion of the random intercept in the model. In the HRVB group, the mean MS-MFR at baseline was 1.07 (95% CI, 0.94-1.22), and after the intervention it was 1.16 (95% CI, 1.06-1.26). In the usual care group, the mean MS-MFR at baseline was 1.20 (95% CI, 1.05-1.38), and at the follow-up visit it was 1.15 (95% CI, 0.94-1.40). We observed no significant increase in log MS-MFR at visit 2 compared with visit 1 in the HRVB group, with a difference of 0.07 units (95% CI, −0.07 to 0.21 units; *P* = .30), or in the usual care group, with a difference of −0.04 units (95% CI, −0.21 to 0.12 units; *P* = .60). There was a statistically significant between-visit difference in the HRVB group (0.06 units; 95% CI, 0.01-0.12 units; *P* = .03) when adjusting for baseline MBF, while there continued to be no between-visit difference in the usual care group. When comparing log MS-MFR between groups, the differences in visit 2 compared with visit 1 were greater in the HRVB than usual care arm by 0.10 units (95% CI, 0.01-0.19 units; *P* = .03). Figure 2 shows MS-MFR levels at the individual level by visit and intervention group to help visualize the patterns described.

**Table 2.** MBF at Rest and at Stress and MS-MFR in the HRVB and Usual Care Groups

Cardiac territory	Outcome, geometric mean (95% CI)			
	HRVB		Usual care	
	Visit 1	Visit 2	Visit 1	Visit 2
LAD artery				
MBF				
At rest	0.84 (0.71-0.98)	0.76 (0.62-0.92)	0.67 (0.54-0.83)	0.70 (0.57-0.85)
At stress	0.90 (0.79-1.02)	0.88 (0.73-1.07)	0.81 (0.64-1.03)	0.80 (0.57-1.13)
MS-MFR^a^	1.07 (0.94-1.23)	1.16 (1.07-1.26)	1.21 (1.05-1.40)	1.15 (0.95-1.40)
LCx artery				
MBF				
At rest	0.90 (0.78-1.03)	0.83 (0.67-1.02)	0.68 (0.56-0.84)	0.73 (0.62-0.86)
At stress	0.96 (0.82-1.14)	0.95 (0.76-1.18)	0.81 (0.64-1.03)	0.84 (0.59-1.19)
MS-MFR^a^	1.08 (0.95-1.22)	1.14 (1.07-1.22)	1.19 (1.04-1.36)	1.15 (0.93-1.43)
RCA				
MBF				
At rest	0.78 (0.66-0.92)	0.70 (0.56-0.89)	0.64 (0.50-0.82)	0.68 (0.56-0.82)
At stress	0.83 (0.71-0.97)	0.82 (0.65-1.02)	0.77 (0.58-1.02)	0.77 (0.55-1.08)
MS-MFR^a^	1.06 (0.95-1.20)	1.16 (1.05-1.28)	1.21 (1.04-1.39)	1.14 (0.95-1.36)
All 3 vessels combined				
MBF				
At rest	0.84 (0.71-0.98)	0.76 (0.61-0.94)	0.66 (0.53-0.83)	0.70 (0.58-0.84)
At stress	0.90 (0.77-1.04)	0.88 (0.71-1.09)	0.80 (0.62-1.02)	0.80 (0.57-1.13)
MS-MFR^a^	1.07 (0.94-1.22)	1.16 (1.06-1.26)	1.20 (1.05-1.38)	1.15 (0.94-1.40)

Abbreviations: HRVB, heart rate variability biofeedback; LAD, left anterior descending; LCx, left circumflex; MBF, myocardial blood flow; MS-MFR, mental stress myocardial flow reserve; RCA, right coronary artery.

^a^ *P* = .03 after adjusting for resting MBF.

Table 3 describes the secondary analysis of myocardial perfusion and LVEF data in a subset of participants. The change between visits in stress sum score was different in the HRVB group (–0.70; 95% CI, –5.55 to 4.15) compared with the usual care group (7.00; 95% CI, –1.33 to 16.07) (*P* = .046), but the other outcomes were not different between groups. No differences were found in the blood pressure, heart rate, and rate pressure product changes with stress between groups at either visit (eTable in Supplement 2).

**Table 3.** Baseline Levels and Changes During Follow-Up in Myocardial Perfusion and LVEF by Group

	HRVB		Usual care		*P* value
	Outcome, mean (95% CI)	Participants, No. (%) (n = 12)	Outcome, mean (95% CI)	Participants, No. (%) (n = 9)	
**Visit 1 only**					
Sum score^a^					
Rest	3.50 (0.42 to 6.58)	9 (75.0)	1.17 (–1.88 to 4.21)	4 (44.4)	.29
Stress	7.48 (3.31 to 11.65)	9 (75.0)	1.83 (–1.56 to 5.23)	4 (44.4)	.07
Stress difference score	3.98 (0.02 to 7.94)	9 (75.0)	0.67 (–1.45 to 2.79)	4 (44.4)	.11
LVEF, %					
Rest	58.40 (50.72 to 66.08)	9 (75.0)	52.63 (45.91 to 59.30)	8 (88.9)	.22
Stress	57.72 (48.77 to 66.67)	9 (75.0)	56.13 (47.37 to 64.88)	8 (88.9)	.77
Stress-rest LVEF difference, pp	0.67 (–3.23 to 4.56)	9 (75.0)	3.50 (–0.27 to 7.27)	8 (88.9)	.24
**Differences between visits**					
Sum score^a^					
Rest	–1.13 (–4.97 to 2.71)	9 (75.0)	4.75 (–2.63 to 12.13)	4 (44.4)	.07
Stress	–0.70 (–5.55 to 4.15)	9 (75.0)	7.00 (–1.33 to 16.07)	4 (44.4)	.046
Stress difference score	0.48 (–2.31 to 3.27)	9 (75.0)	2.25 (–2.71 to 7.21)	4 (44.4)	.42
LVEF, pp					
Rest	2.65 (–2.83 to 8.13)	9 (75.0)	–1.19 (–4.23 to 1.86)	8 (88.9)	.21
Stress	4.22 (–0.96 to 9.41)	9 (75.0)	–1.25 (–7.59 to 5.09)	8 (88.9)	.11
Stress-rest LVEF difference, pp	0.78 (–4.31 to 5.87)	9 (75.0)	0.06 (–4.64 to 4.51)	8 (88.9)	.78

Abbreviations: HRVB, heart rate variability biofeedback; LVEF, left ventricular ejection fraction; pp, percentage points.

^a^ Score range 0 to 68, with higher scores indicating more perfusion defects.

There were 2 participants in the HRVB group for whom MS-MFR decreased contrary to the group mean; at least 1 of each participant’s scans had suboptimal image quality. In addition, marked hemodynamic changes were observed in 2 cases with unusually high MS-MFR values—1 in a usual care participant at visit 2 and 1 in an HRVB participant at visit 1.

## Discussion

In this pilot study of patients with CAD, we demonstrated a statistically significant effect of HRVB on MBF. Specifically, participants in the HRVB group showed increases in MS-MFR after the intervention compared with usual care. To our knowledge, this is the first study of its kind to examine an intervention aimed at improving MBF regulation after a mental stress challenge.

This study is complemented by other studies that have examined the effects of HRVB on cardiac risk factors. Previous literature has focused on outcomes other than MFR, including HRV,^21,22^ blood pressure,^23^ psychological stress (depression, anxiety),^24^ and hospitalizations in patients with CAD.^21^ We were able to further elucidate the health benefits of HRVB and examine its potential role in improving the coronary physiologic response to acute stress.

The between-group difference in stress perfusion, which was a secondary outcome, was also significant, although the sample size was limited. The trends observed suggest the need for more research in a larger study. No significant differences in hemodynamic effects were observed, and more research is needed to understand these negative results as well.

### Limitations

Our study findings are subject to limitations. The small sample size and group imbalances may increase the likelihood of spurious findings, and we had a limited sample size to adjust for all of them. Nonetheless, we adjusted for resting MBF to help account for these baseline differences. The findings are not generalizable to individuals without CAD. We did not examine HRV as an outcome in this analysis, which limits our ability to compare our intervention with other HRVB studies that used HRV as an outcome. The implications of improving MS-MFR with HRVB as a way to reduce mortality risks are not clear and warrant further research as well.

We also noted outliers in which the changes between visits were opposite of the overall group trend. For the 2 participants in the HRVB group for whom MS-MFR decreased contrary to the group mean, at least 1 of their scans had suboptimal image quality, which may have increased the likelihood of measurement error. Additionally, in the 2 cases with unusually high MS-MFR values (1 at visit 2 in a usual care participant and 1 at visit 1 in an HRVB participant), marked hemodynamic changes were also observed and may have contributed to the elevated MS-MFR. These factors, which were not clearly related to the intervention, likely biased the results toward the null and highlight methodologic considerations for future studies building on this investigation.

## Conclusions

In this randomized clinical trial of participants with CAD, we found a significant effect of HRVB on MS-MFR that warrants further exploration in a larger clinical trial. Although the results were statistically significant, a larger study should examine whether these findings are reproducible and scalable.

## Notes

Supplement 1.
Trial Protocol

Supplement 2. **eTable.** Hemodynamic Changes by Visit in the HRVB vs Usual Care Arm

Supplement 3.
Data Sharing Statement
