# Supplementary material for: Heart Rate Variability Biofeedback and Mental Stress Myocardial Flow Reserve: A Randomized Clinical Trial
Source: JAMA Netw Open. 2025 Oct 21;8(10):e2538416. doi: 10.1001/jamanetworkopen.2025.38416 (PMC12541537; doi:10.1001/jamanetworkopen.2025.38416)
Supplement: Supplement 2. — eTable. Hemodynamic Changes by Visit in the HRVB vs Usual Care Arm [file jamanetwopen-e2538416-s002.pdf]

Supplementary Online Content

Shah AJ, Raggi P, She H, et al. Heart rate variability biofeedback and mental stress myocardial flow reserve: a randomized clinical trial. *JAMA Netw Open*. 2025;8(10):e2538416. doi:10.1001/jamanetworkopen.2025.38416

eTable. Hemodynamic Changes by Visit in the HRVB vs Usual Care Arm

This supplementary material has been provided by the authors to give readers additional information about their work.

© 2025 Shah AJ et al. *JAMA Network Open*.

eTable. Hemodynamic Changes by Visit in the HRVB versus Usual Care Arm. All values are geometric means and 95% confidence intervals are provided in parentheses. The p value is the statistical test for the treatment effects on the difference between visit 1 and visit 2

Outcome	HRVB Visit 1	HRVB Visit 2*	Usual Care Visit 1	Usual Care Visit 2*	p
**Min SBP at Rest (mmHg)**	132.4 (114.8, 150.0)	131.6 (116.4, 146.8)	119.9 (106.3, 133.4)	120.3 (108.5, 132.0)	0.92
**Min DBP at Rest (mmHg)**	71.0 (63.9, 78.0)	69.5 (63.1, 75.9)	61.8 (57.1, 66.5)	63.1 (58.0, 67.8)	0.70
**Min HR at Rest (BPM)**	58.1 (51.9, 64.2)	60.0 (55.3, 64.7)	52.5 (50.0, 55.1)	54.4 (50.3, 58.6)	0.70
**Max SBP at Stress (mmHg)**	160.2 (143.5, 177.0)	154.4 (134.5, 174.3)	149.5 (134.6, 164.4)	137.7 (125.4, 150.0)	0.28
**Max DBP at Stress (mmHg)**	78.6 (69.8, 87.4)	78.7 (69.6, 87.8)	75.5 (70.2, 80.8)	71.7 (65.2, 78.2)	0.18
**Max HR at Stress (BPM)**	71.8 (63.2, 78.8)	71.9 (65.1, 78.6)	68.6 (54.7, 75.4)	65.2 (52.5, 78.7)	0.14
**RPP Rest Min (mmHg*BPM)**	7499 (6327, 8890)	7757 (6766, 8893)	6150 (5082, 7442)	6406 (5170, 7936)	0.80
**RPP Stress Max (mmHg*BPM)**	11087 (9126, 13470)	10787 (9019, 12901)	9889 (7549, 12954)	8776 (6903, 11158)	0.11
**RPP Reactivity (mmHg*BPM)**	3099 (1939, 4954)	2615 (1505, 4546)	3373 (1923, 5918)	2081 (1194, 3627)	0.31

RPP=rate pressure product. SBP=systolic blood pressure. DBP=diastolic blood pressure. Min=minimum. Max=maximum.

© 2025 Shah AJ et al. *JAMA Network Open*.
