# Supplementary material for: Heart Rate Variability Biofeedback and Mental Stress Myocardial Flow Reserve: A Randomized Clinical Trial
Source: JAMA Netw Open. 2025 Oct 21;8(10):e2538416. doi: 10.1001/jamanetworkopen.2025.38416 (PMC12541537; doi:10.1001/jamanetworkopen.2025.38416)
Supplement: Supplement 3. — Data Sharing Statement [file jamanetwopen-e2538416-s003.pdf]

Data Sharing Statement

Data
Additional Information: NCT02657382
Data available: No

Additional Information
Explanation for why data not available: We may share data upon reasonable request and data use agreement
